# Genetic disorders of neurotransmitter release machinery

**DOI:** 10.3389/fnsyn.2023.1148957

**Published:** 2023-03-31

**Authors:** Burak Uzay, Ege T. Kavalali

**Affiliations:** ^1^Vanderbilt Brain Institute, Nashville, TN, United States; ^2^Department of Pharmacology, Vanderbilt University, Nashville, TN, United States

**Keywords:** SNAREopathy, synapse, neurogenetic disorders, SNAP25, synaptobrevin, syntaxin, synaptotagmins, Munc13

## Abstract

Synaptic neurotransmitter release is an evolutionarily conserved process that mediates rapid information transfer between neurons as well as several peripheral tissues. Release of neurotransmitters are ensured by successive events such as synaptic vesicle docking and priming that prepare synaptic vesicles for rapid fusion. These events are orchestrated by interaction of different presynaptic proteins and are regulated by presynaptic calcium. Recent studies have identified various mutations in different components of neurotransmitter release machinery resulting in aberrant neurotransmitter release, which underlie a wide spectrum of psychiatric and neurological symptoms. Here, we review how these genetic alterations in different components of the core neurotransmitter release machinery affect the information transfer between neurons and how aberrant synaptic release affects nervous system function.

## 1. Introduction

**FIGURE 1:**
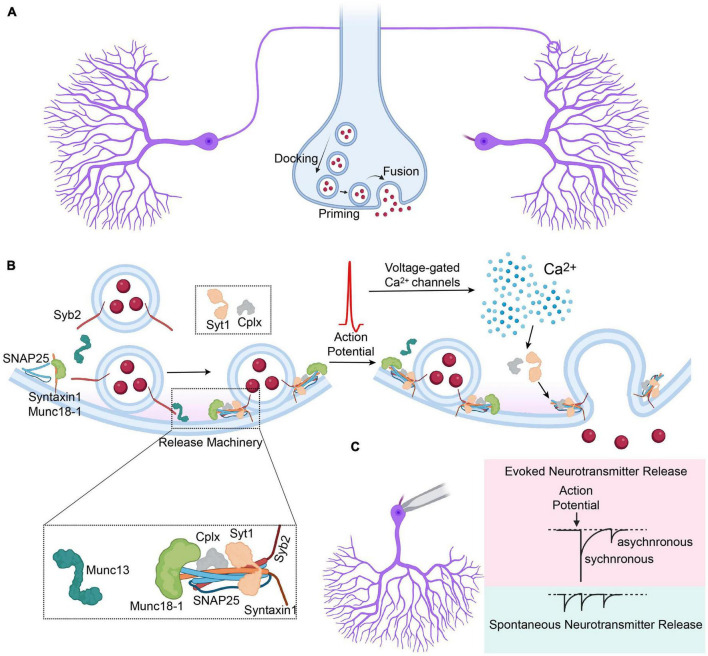
Overview of the synaptic vesicle release. **(A)** Synaptic vesicles are docked to the presynaptic terminal, followed by priming and fusion that is mediated by the components of the synaptic release machinery. **(B)** Graphical depiction of synaptic vesicle release induced by the arrival of an action potential to the presynaptic terminal, preceded by priming of the docked synaptic vesicles. Cplx, complexin; Syt1, synaptotagmin-1; Syb2, synaptobrevin-2. **(C)** Graphical depiction representing different modes of synaptic vesicle release and their representative electrophysiological traces, that comprise evoked (synchronous and asynchronous) and spontaneous release.

Synaptic neurotransmission depends on precisely orchestrated neurotransmitter release mediated by highly specialized proteins, which form the components of the release machinery. Synaptic neurotransmitter release takes place by the fusion of synaptic vesicles with the presynaptic plasma membrane, which is primarily mediated by soluble N-ethylmaleimide-sensitive factor attachment protein receptors (SNARE) proteins (Hu et al., 2003; Jahn and Fasshauer, 2012). At mammalian central nervous system synapses, SNARE proteins consist of syntaxin-1 and SNAP25 on the plasma membrane, also known as t-SNAREs, and synaptobrevin-2 (Syb2, also called VAMP2) on the synaptic vesicle, also known as the v-SNARE (Chen et al., 1999). The v- and t-SNAREs assemble from N termini toward C termini, *via* a zippering process that pulls the membranes together and drives vesicle fusion. This process is tightly regulated by a number of other presynaptic proteins, such as Munc18 and Munc13, Ca^2+^ sensing synaptotagmins and complexins (Brunger, 2005; Rizo and Rosenmund, 2008; Figures 1A, B).

On the presynaptic membrane, syntaxin-1 is assembled with Munc18-1 in a closed configuration that does not permit SNARE complex assembly (Dulubova et al., 1999; Toonen and Verhage, 2007). This closed configuration is disinhibited by Munc13-1 upon activation by RIM proteins allowing trans-SNARE complex formation and consequent vesicle priming (Betz et al., 2001; Dulubova et al., 2005; Ma et al., 2011). The docking and priming of synaptic vesicles are completed when Syb2 forms a complex with syntaxin-1 and SNAP25 followed by complexin binding to the assembled SNARE complex, which provides stabilization by preventing de-priming (Chen et al., 2002; Rizo and Xu, 2015). Upon arrival of an action potential to the presynaptic terminal, the rapid increase in presynaptic calcium through presynaptic voltage gated Ca^2+^ channels is sensed by synaptotagmin-1, which drives the zippering of the SNARE complex and the fusion of the membranes (Südhof, 2013). Release of the synaptic vesicles does not only take place upon the arrival of an action potential to the presynaptic terminal, but also takes place spontaneously, which has significant physiologic implications (Figure 1C; reviewed in Kavalali, 2015; Chanaday and Kavalali, 2018; Guzikowski and Kavalali, 2021).

Mutations in the components of this complex machinery perturb synaptic vesicle release, which could selectively disrupt microcircuits in different brain regions, excitatory-inhibitory balance and could eventually result in neuronal death (reviewed in Verhage and Sørensen, 2020). Perturbations in the synaptic vesicle release due to mutations in different components of the release machinery clinically present themselves as a wide array of symptoms, usually with an early onset (Saitsu et al., 2008; Hamdan et al., 2017; Baker et al., 2018; Heyne et al., 2018). Most patients present symptoms in the first year of life with neurodevelopmental delays in language, cognitive and motor milestones and infantile hypotonia. In addition, patients may present with seizures, ataxia, movement disorders (i.e., hyperkinesia), behavioral (i.e., autism), or ophthalmic symptoms (i.e., cone/rod dystrophy, strabismus) (Johnson et al., 2003; Baker et al., 2018; Verhage and Sørensen, 2020). Due to significant heterogeneity in clinical presentation, patients who have mutations in the synaptic release machinery receive various clinical diagnoses including the Ohtahara syndrome, the West syndrome, non-syndromic epilepsy, autism, Rett-like syndrome, or developmental and epileptic encephalopathies (DEE). In the recent years, a new diagnostic category, “SNAREopathies,” was proposed, in order to solve this diagnostic odyssey and to unify the efforts to understand the pathophysiology of different SNARE variants (Verhage and Sørensen, 2020).

Currently, the treatment options are limited for the patients suffering from SNAREopathies and the treatment is mainly focused on symptomatic relief to increase the patients’ quality of life. Additional preclinical studies are critical in increasing our understanding of the molecular and cellular effects of specific SNARE variants to develop potent treatment options. Here, we reviewed the clinical reports and the preclinical studies investigating the effects of different pathogenic variants of the synaptic release machinery on vesicle recycling and synapse ultrastructure. We utilized Uniprot, DECIPHER, ClinVar, OMIM, and gnomAD databases to identify the pathogenic (or likely pathogenic) variants that fits ACMG (American College of Medical Genetics and Genomics) criteria, followed by an in-depth literature search to extract the clinical and preclinical data that provides functional implications of specific variants on the release machinery.

## 2. Genetic disorders of SNARE proteins

### 2.1. Synaptobrevin-2

**FIGURE 2:**
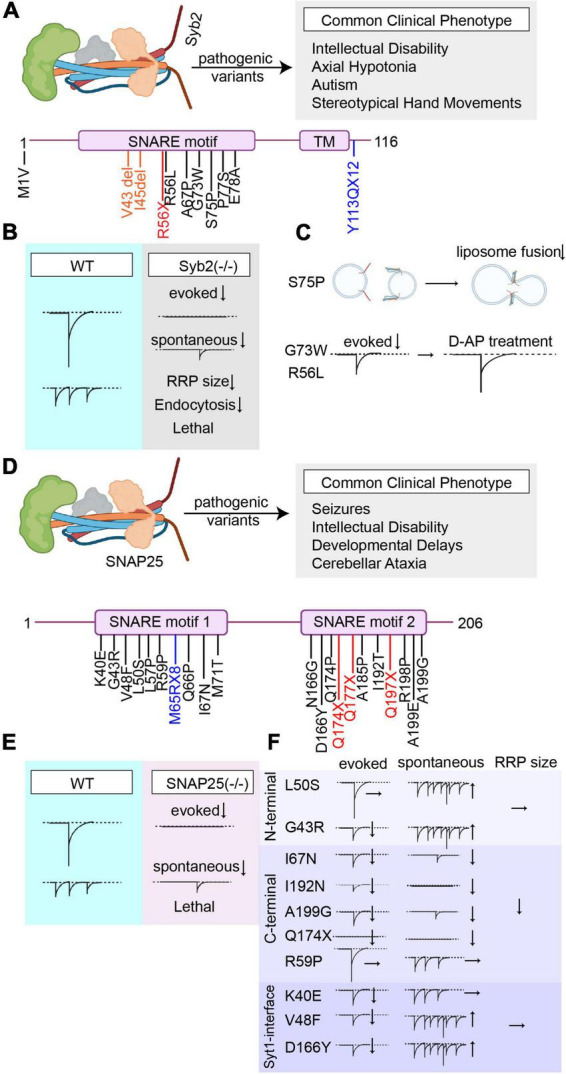
Overview of the pathogenic Syb2 and SNAP25 variants’ effects on synaptic vesicle release. **(A)** Summary of all reported pathogenic Syb2 (NCBI Accession #: AAF15551.1) variants and the most common clinical phenotype of the patients harboring these variants (Variants are color-coded based on their effects on the protein. Nonsense mutations are presented in red, missense mutations are presented in black, frameshift mutations are presented in blue, and deletions/insertions are presented in orange). **(B)** Graphical depiction demonstrating the changes in synaptic vesicle release upon genetic deletion of Syb2. **(C)** Graphical summary of the findings explaining aberrant forms of vesicle fusion and neurotransmission caused by different Syb2 variants (S75P, G73W, and R56L). **(D)** Summary of all reported pathogenic SNAP25 (NCBI Accession #: NP_001309838.1) variants and the most common clinical phenotype of the patients harboring these variants (Variants are color-coded based on their effects on the protein. Nonsense mutations are presented in red, missense mutations are presented in black, and frameshift mutations are presented in blue). **(E)** Graphical depiction demonstrating the changes in synaptic vesicle release upon genetic deletion of SNAP25. **(F)** Graphical summary of the findings explaining the effects of different SNAP25 variants (N-terminal, C-terminal, or Syt1-interface variants) on different modes of neurotransmitter release.

Synaptobrevin-2 is a vesicular SNARE protein that forms the core of the SNARE complex with syntaxin-1 and SNAP25 and is a critical component of the machinery mediating synaptic vesicle fusion and neurotransmitter release (Figure 2A). Syb2 knockout mice die immediately after birth and show significantly impaired neurotransmitter release (Schoch et al., 2001). Neurons lacking Syb2 demonstrate a significant reduction in spontaneous release, the number of docked vesicles and the readily releasable pool (RRP) size and almost complete loss of evoked release (Schoch et al., 2001; Deák et al., 2006; Imig et al., 2014; Zimmermann et al., 2014; Figure 2B). In addition to its critical role in vesicle exocytosis, Syb2 also plays an important role in the rapid endocytosis, which is required for the replenishment of synaptic vesicles for consecutive events and Syb2 knockout neurons show significant impairments in stimulus-dependent endocytosis (Deák et al., 2004; Chanaday and Kavalali, 2021). Although the absence of Syb2 is lethal, heterozygous Syb2 (+/−) mice only show a mild behavioral phenotype, most likely due to genetic redundancy (Monteggia et al., 2019).

To date, there are 11 pathogenic (or likely pathogenic) variants that are reported in the Syb2 gene, that comprise 7 missense (S75P, E78A, P77S, G73W, R56L, M1V, and A67P), 1 nonsense (R56X), and 1 frameshift (Y113QfsX12) mutation, in addition to 2 single amino acid deletions (V43del and I45del). Patients who harbor these pathogenic variants commonly present with intellectual disability, axial hypotonia, autism, and Rett syndrome-like features (stereotyped hand movements), symptoms that are in line with the extrapolation of the microarray data showing the highest expression of Syb2 in frontal lobes and putamen (Salpietro et al., 2019; Figure 2A).

In 2019, the first five patients that harbor pathological Syb2 variants were identified, which presented with a neurodevelopmental disorder characterized by axial hypotonia, intellectual disability, autism and Rett syndrome-like features (stereotypical and repetitive movements). Among these variants, there were three missense mutations (S75P, E78A, and P77S) and two single amino acid deletions (V43del and I45del), which were all in the SNARE motif of Syb2 (C-terminal). Missense mutations resulted in a more severe clinical phenotype compared to single amino acid deletions and presented with additional neurological symptoms such as movement disorders (choreic movements) and central visual impairments. These pathological C-terminal variants also caused seizures and/or abnormalities in the EEG (Salpietro et al., 2019). Lipid-mixing assays, performed using some of these Syb2 variants showed a fusion defect for S75P mutation compared to wild-type (WT) Syb2. The variants effecting Ser75 have been previously shown to impair the interaction of Syb2 with Munc18-1 and cause a decrease in the stimulatory effects of Munc18-1 in lipid-mixing assays, where the inclusion of Munc18-1, normally, facilitates SNARE assembly and membrane fusion through the C-terminal region of Syb2 (Sørensen et al., 2002; Shen et al., 2007, 2015). In line with previous studies, inclusion of Munc18-1 in lipid-mixing using S75P variant failed to increase membrane fusion, indicating a profound loss-of-function (Salpietro et al., 2019; Figure 1C). In 2020, another six unrelated patients were reported that harbor *de novo* pathogenic variants in the Syb2 gene (Simmons et al., 2020; Sunaga et al., 2020). Among these six novel variants, there were three missense mutations (G73W, R56L, and A67P), one frameshift mutation that creates a premature stop codon (Y113QfsX12), one missense mutation that eliminates the initiator methionine (M1V), and one nonsense mutation (R56X) that results in premature truncation of Syb2. These patients commonly presented with global developmental delay, autism, and behavioral abnormalities and a higher propensity to develop epilepsy (Simmons et al., 2020; Sunaga et al., 2020). In addition to that, they presented with an array of psychiatric symptoms ranging from hallucinations, delusions, anxiety, depression (R56X) to obsessive-compulsive disorder (OCD) tendencies (G73W), and attention-deficit/hyperactivity disorder (Y113QfsX12 and M1V).

Since most of these variants affect the SNARE motif of Syb2, one would expect perturbations in SNARE complex formation and impaired synaptic vesicle fusion precipitated by these variants, which is supported by lipid-mixing studies (Salpietro et al., 2019; Figure 2C). However, how the majority of these pathogenic Syb2 variants alter the neuronal functions and different modes of synaptic vesicle release is currently unknown. The effects of only some variants (G73W, R56L, and R56X) on synaptic release was investigated using live-cell imaging and electrophysiology. The live-cell imaging experiments showed that the neurons carrying G73W and R56L variants had a slower rate of exocytosis in response to stimulation, although R56X variant did not significantly alter the rate of exocytosis. In line with the imaging results, whole-cell voltage clamp recordings showed that G73W, R56L variants resulted in decreased evoked release, while R56X did not show a significant phenotype. The release probability was also found to be significantly decreased in the synapses that have G73W or R56L variants (Simmons et al., 2020). These findings underscore the significant variability in functional outcomes caused by different Syb2 variants and emphasize the need for additional research to determine the variant-specific effects on presynaptic functions. How different Syb2 variants alter different modes of vesicle release is a critical question to be answered by future research, which would potentially open up new venues in identifying the roles of Syb2 in neural circuits and in developing treatment options for the patients suffering from Syb2-associated neurodevelopmental disorders. The current treatment options are very limited for the patients harboring pathogenic Syb2 variants and mostly aim symptomatic relief. Boosting presynaptic Ca^2+^ to relieve the defects in exocytosis was proposed as a potential treatment strategy (Simmons et al., 2020). This premise was tested with a K^+^ channel inhibitor, D-AP (D-aminopyridine), that prolongs action potentials and therefore increases local Ca^2+^ availability. When treated with D-AP, the rate and extent of exocytosis of synaptic vesicles significantly increased in neurons transfected with R56X, G73W, and R56L variants. Whole-cell voltage clamp recordings showed that D-AP treatment switches the mode of release from fast synchronous to asynchronous, therefore increasing the total synaptic charge transfer ameliorating synaptic release deficits. Extrapolating from this *in vitro* data, 4-AP (4-aminopyridine), a drug clinically approved for multiple sclerosis, was begun as an off-label treatment in a patient that has R56X variant and the treatment resulted in dramatic improvement within 2 months. The comparison of neuropsychological tests pre- and post-treatment with D-AP demonstrated a significant improvement in cognitive and social abilities (Simmons et al., 2020; Figure 2C). Overall, current evidence suggests that increasing presynaptic Ca^2+^ is a viable strategy that warrants further investigation. The use of aminopyridines could be a potential treatment option to rescue synaptic release deficits in SNAREopathies in patients that do not suffer from epilepsies, and they should be cautiously used given their high risk of lowering the seizure threshold (Heuzeroth et al., 2019).

### 2.2. Synaptosomal-associated protein of 25 kDa (SNAP25)

Synaptosomal-associated protein of 25 kDa (SNAP25) is a SNARE protein on the plasma membrane that mediates synaptic neurotransmitter release by forming a SNARE complex with syntaxin-1 and Syb2 (Rizo and Rosenmund, 2008; Figure 2D). In addition to its major role in fast exocytosis, SNAP25 also plays a role in slow clathrin-coated endocytosis of hippocampal synapses, in vesicle-docking and priming as well as modulation of voltage gated calcium channels during intense activity (Pozzi et al., 2008; Mohrmann et al., 2013; Zhang et al., 2013). SNAP25 has a ubiquitously expressed homolog SNAP23, which plays a role in asynchronous release and two splice variants SNAP25a and SNAP25b, which are highly homologous to each other, only differ by nine amino acid residues that correspond to alternative splicing of exon 5 (Bark et al., 1995; Weber et al., 2014). SNAP25a and SNAP25b are differentially expressed during the development of brain where SNAP25a is more predominantly expressed during the embryogenesis and SNAP25b is more predominantly expressed in the adult brain (Bark et al., 1995). Moreover, SNAP25b is enriched in the nerve terminal plasma membranes of central synapses or motor endplates whereas SNAP25a is more abundant in cytoplasm and nerve tracts (Prescott and Chamberlain, 2011; Yamamori et al., 2011).

Knockout of SNAP25 results in neonatal lethality due to respiratory failure caused by failed evoked release at the phrenic nerve terminals that innervate the diaphragm, thereby preventing newborn pups from breathing (Washbourne et al., 2002). SNAP25 knockout neurons show significant impairments in evoked release whereas spontaneous release continues to take place although at a decreased rate reflecting a decrease in the number of functional synapses (Washbourne et al., 2002; Figure 2E). In contrast to *in vivo* findings showing that knockout of SNAP25 does not affect neuronal survival or arborization of neurites, *in vitro* deletion of SNAP25 disturbs neuronal survival and axonal arborization, in addition to its deleterious effects on evoked release (Bronk et al., 2007; Delgado-Martínez et al., 2007; Hoerder-Suabedissen et al., 2018). Lentiviral expression of SNAP25a and SNAP25b in these neurons was shown to rescue these impairments where SNAP25b functions superior to SNAP25a in terms of vesicle priming, which uses a larger vesicle pool, as expected, upon synaptic maturation (Delgado-Martínez et al., 2007). SNAP25b deficient mice, with preserved expression of SNAP25a, demonstrated spontaneous seizures, hyperactivity, anxiety-like behavior, and impairments in short-term synaptic plasticity underscoring the importance of SNAP25b in neural circuitry (Johansson et al., 2008). Since the homozygous knockout of SNAP25 results in embryonic lethality, it precludes investigation of the loss-of-function effects of this protein in the central nervous system. The haploinsufficiency caused by heterozygous deletion of SNAP25 in SNAP25(+/−) mice results in decreased ambulation in open field test without any other abnormalities in behavioral tests assessing anxiety-like behavior, learning, and memory or prepulse inhibition (Monteggia et al., 2019). Although the behavior is not profoundly affected, a decreased threshold for kainate-induced seizures is observed in SNAP25(+/−) mice, while they do not have spontaneous seizures (Corradini et al., 2014). Electrophysiological investigation of SNAP25(+/−) hippocampal slices and primary hippocampal cultures reveal that neither spontaneous release nor the overall efficiency of evoked release is affected by haploinsufficiency except a small decrease in release probability (Alten et al., 2021). Majority of patients that harbor mutations in the SNAP25 gene are heterozygous and lack a normal SNAP25 allele. The studies investigating the heterozygous loss of SNAP25 point out that haploinsufficiency is unlikely the disease mechanism, given the severity of the clinical symptoms, nevertheless it might be a factor aggravating the aberrant synaptic transmission precipitated by individual SNAP25 variants (Alten et al., 2021).

To date, there are 24 reported pathogenic (or likely pathogenic) variants of SNAP25, which comprise 18 missense mutations(G43R, L50S, V48F, R59P, K40E, L57P,L57R, Q66P, I67N, M71T, N166G, D166Y, Q174P, A185P, I192T, R198P, A199E, and A199G), three nonsense mutations (Q177X, Q174X, and Q197X), one frameshift mutation (M64RfsX8) and two splice donors (c.72 + 1G > A and c.114 + 2T > G). The patients harboring pathogenic SNAP25 variants commonly present with a wide array of neurological symptoms including seizures, various degrees of intellectual disability, muscle weakness, speech delays, and cerebellar ataxia (Figure 2D). The clinical phenotypes of these patients are commonly categorized under the clinical diagnosis of “developmental and epileptic encephalopathies (DEE).” DEE is a group of conditions characterized by developmental regression, intellectual disability and frequent epileptiform activity (Hamdan et al., 2017). Among DEEs caused by SNAP25 variants, R59P variant results in the mildest phenotype whereas the Q174X and I192N variants result in most severe phenotype and lethality in the first year of life (Deciphering Developmental Disorders Study, 2017; Fukuda et al., 2018; Alten et al., 2021). This variability in clinical phenotype could be explained by variant specific perturbations in synaptic vesicle release, precipitated either by impaired SNARE complex formation resulting in a loss-of-function phenotype or aberrant forms of vesicle release that demonstrate a dominant negative effect (Alten et al., 2021).

In 2013, the first pathogenic variant in the SNAP25 gene was reported, caused by a missense mutation, V48F, in a patient presenting with severe encephalopathy, intellectual disability and generalized epilepsy that is resistant to antiepileptic (AED) treatment. The patient had a history of infantile hypotonia and an early-onset generalized seizures that began at 5 months of age (Rohena et al., 2013). In 2014, another missense mutation, I67N, was reported in SNAP25b which presented with intellectual disability, myasthenia, cerebellar ataxia, severe speech delay, and intractable seizures. The neuromuscular transmission at the motor endplates were significantly affected due to reduced evoked quantal release (Shen et al., 2014). Lipid-mixing studies showed that I67N variant significantly decreased Ca^2+^ triggered fusion of liposomes. In addition, amperometric assessment of SNAP25 I67N transfected bovine chromaffin cells show significantly decreased evoked catecholamine release compared to wild-type SNAP25 transfected cells (Shen et al., 2014), underscoring the severe perturbations in exocytosis caused by SNAP25 I67N, in addition to its dominant negative effects (Rebane et al., 2018). In the last decade, upon the more widespread use of whole exome sequencing in determining the genetic origins of neuropsychiatric diseases of unexplained origin, many additional pathogenic variants in the SNAP25 gene were identified, presenting with severe neurological symptoms (Deciphering Developmental Disorders Study, 2017; Hamdan et al., 2017; Fukuda et al., 2018; Heyne et al., 2018). In a recent cohort of patients that harbor heterozygous missense or loss-of-function variants in SNAP25 gene, a thorough phenotyping was performed, in addition to a molecular *in silico* modeling to predict functional implications of these variants. From 23 individuals that were enrolled in this study, 15 missense variants and four loss-of-function (two nonsense and two splice donor variants) were identified. These pathogenic missense variants were either in highly conserved t-SNARE homology domain 1 or homology domain 2. Seizures were reported in the majority of individuals that are resistant to antiepileptic drug (AED) treatment and the most common seizure type was generalized tonic-clonic seizure (Klöckner et al., 2021). The most common neurological finding was hypotonia and although most individuals did not show behavioral symptoms, some showed signs of autism. Among these clinical phenotypes, the missense mutation G43R showed the mildest phenotype with mild to moderate ID and isolated generalized seizures that respond to AED treatment whereas the individuals with nonsense variants Q174X and Q197X showed the most severe clinical phenotypes. *In silico* structural modeling of SNAP25 pathogenic variants revealed that some of these variants could be destabilizing the structure of SNAP25 by causing steric clashes (G43R, L57R, Q66P, and Q174P), by weakening intramolecular interactions (L50S, K40E, and I192T) or by enhancing backbone flexibility (N166G, A199G, and Q197X). Some of these variants are predicted to impair the interactions of SNAP25 with other SNARE components like syntaxin-1 (I67N), or Syb2 (A199G). Another group of variants are also predicted to impair the interaction of SNAP25 to SNAPα (V48F and D166Y) and some variants have multiple affects in impairing both the SNAP 25 structure and its interactions with other proteins (G43R and M71T) (Klöckner et al., 2021). The effects of some of these SNAP25 variants on different modes synaptic release was investigated in a recent study through electrophysiological assessment of primary hippocampal neurons (Alten et al., 2021). In that study, authors categorized 10 different SNAP25 variants according to the structural (and functional) protein subdomains where amino acid substitutions take place (or termination for Q174X) and included L50S, G43R variants in the N-terminal group; I67N, I192N, and A199G variants in the C-terminal group; and R59P, Q174X, K40E, V48F, and D166Y variants in the synaptotagmin-1 (Syt-1)-interface group. Circular dichroism (CD)-spectroscopy showed that although Syt-1 interface variants did not have a significant effect on SNARE complex stability compared to WT controls, both N-terminal and C-terminal variants resulted in significant SNARE complex instability. This is consistent with the structure of the SNARE complex core where both N- and C-terminals of SNAP25 mediate the hydrophobic interactions in SNARE complex formation whereas the Syt-1 interface is facing outwards (Alten et al., 2021). Electrophysiological assessment of hippocampal neurons transduced with these variants reveal that the RRP size was not altered with Syt-1 interface or N-terminal variants whereas all C-terminal variants significantly reduced the RRP size, underlining the critical role of the C-terminal in maintaining the RRP. Both Syt1-interface variants K40E, V48F, and D166Y; and C-terminal variants I67N, Q174X, I192N, and A199G perturbed evoked release, most likely by impairing Syt-1 interaction of SNAP25 and impairing the role of the C-terminal of SNAP25 as the power stroke for fusion, respectively. N-terminal variant G43R mildly impaired evoked release whereas both N-terminal variant L50S and C-terminal variant R59P did not have a significant effect on evoked release. Authors also investigated the individual effects of these variants on spontaneous neurotransmitter release, to test whether changes in spontaneous release could explain the significant clinical heterogeneity caused by different variants. Among Syt-1 interface variants, V48F and D166Y significantly increased spontaneous vesicle fusion whereas K40E did not have a significant effect compared to WT controls. Ca^2+^ chelation by BAPTA-AM showed that this increase in spontaneous vesicle fusion frequency was not a calcium-sensitive process, and resulted due to an intrinsic increased propensity of spontaneous fusion. Expression of N-terminal variants G43R and L50S also increased spontaneous vesicle fusion frequency, which could result by an indirect alteration of Syt-1 interaction and consecutive unclamping of spontaneous release. Expression of C-terminal variants in SNAP25 knockout cultures failed to rescue spontaneous release in addition to a significantly perturbed evoked release, providing evidence that the C-terminal constitutes the force for membrane fusion regardless of the mode of release (Alten et al., 2021; Figure 2F). These variant-specific disturbances in synaptic transmission could have additional effects on the network activity and overall excitability. Authors tested this premise by employing whole-cell current clamping and show that disturbances in synaptic release perturb both action potential firing pattern and the resting membrane potential. Variants that caused more than four-fold increase in spontaneous vesicle release (V48F, L50S, and D166Y) resulted in a more depolarized resting membrane potential with prolonged action potential kinetics. On the other hand, the C-terminal variants that impair both modes of neurotransmitter release resulted in significant silencing of the network activity. Moreover, the degree of the disturbances in the network activity correlated with the degree of clinical phenotypes seen in patients harboring these variants. This study underscores that variants in the release machinery can alter spontaneous release, in addition to their effects on evoked release and showed that dysregulation of spontaneous release alone could be sufficient to cause SNAREopathies (i.e., L50S variant). This might explain the clinical diversity caused by different variants and the treatment-resistant nature of SNAREopathies. It is necessary to generate novel treatment approaches targeting spontaneous neurotransmitter release for patients harboring variants that selectively alter spontaneous neurotransmission. This premise should also be tested for different SNARE mutations to thoroughly elucidate the pathophysiology of different SNARE variants, to provide personalized, variant-specific treatments (Alten et al., 2021).

### 2.3. Syntaxin-1

**FIGURE 3:**
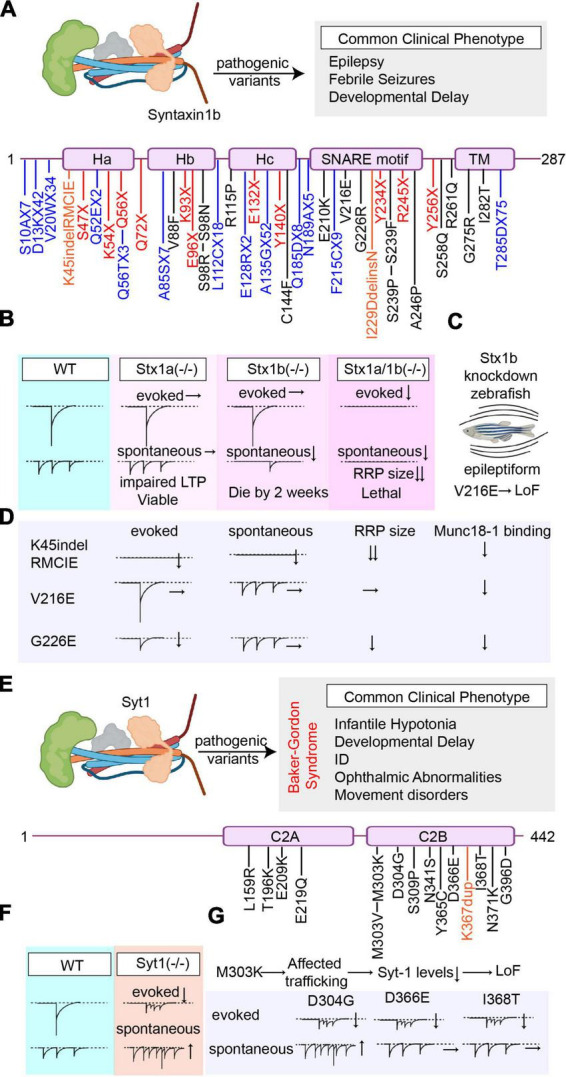
Overview of the pathogenic Stx1b and Syt1 variants’ effects on synaptic vesicle release. **(A)** Summary of all reported pathogenic Stx1b (NCBI Accession #: NP_443106.1) variants and the most common clinical phenotype of the patients harboring these variants (Variants are color-coded based on their effects on the protein. Nonsense mutations are presented in red, missense mutations are presented in black, frameshift mutations are presented in blue, and deletions/insertions are presented in orange). **(B)** Graphical depiction demonstrating the changes in synaptic vesicle release upon genetic deletion of Stx1a and/or Stx1b. **(C)** Graphical depiction showing that the epileptiform phenotype caused by Stx1b-knockdown in zebrafish cannot be rescued by the V216E variant indicating loss-of-function. **(D)** Graphical summary of the findings explaining the effects of different Stx1b variants (K45indel, V216E, and G226E) on different modes of neurotransmitter release and their mechanism of action. **(E)** Summary of all reported pathogenic Syt1 (NCBI Accession #: AAH58917.1) variants and the most common clinical phenotype of the patients harboring these variants (Variants are color-coded based on their effects on the protein. Missense mutations are presented in black, and duplications are presented in orange). **(F)** Graphical depiction demonstrating the changes in synaptic vesicle release upon genetic deletion of Syt1. **(G)** Graphical summary of the findings explaining the effects of different Syt1 variants (D4G, D366E, I368T, and M303K) on different modes of neurotransmitter release.

Syntaxin-1 (Stx1) is a plasma membrane-bound presynaptic protein and a core component of the SNARE complex that mediates synaptic vesicle fusion (Südhof and Rothman, 2009; Chanaday and Kavalali, 2018; Figure 3A). Syntaxin-1 has two main paralogs, Stx1a and Stx1b, that are commonly co-expressed in the central nervous system (Ruiz-Montasell et al., 1996). Their expression pattern differs in the peripheric nervous system where Stx1a is more predominantly expressed in the nerve terminals of sensory neurons reaching the spinal cord and Stx1b predominates at motor endplates (Aguado et al., 1999).

The double knockout of Stx1a and Stx1b in mice shows postnatal lethality and neurons lacking both Stx1a and 1b show rapid degeneration, underlining the role of syntaxin-1 in neuronal survival (Vardar et al., 2016; Feringa et al., 2023). Electrophysiological and ultrastructural investigation of primary neuronal cultures of Stx1a/1b double knockout mice show that both spontaneous and evoked neurotransmission are significantly perturbed, with a decrease in docked vesicle number and the RRP size (Imig et al., 2014; Mishima et al., 2014; Vardar et al., 2016). When Stx1a is knocked out alone, the knockout mice are viable and only show a mild phenotype with learning and memory deficits, indicating that Stx1a is not essential for neuronal survival but could play a role in synaptic plasticity (Fujiwara et al., 2006; Gerber et al., 2008). Electrophysiological investigation of Stx1a knockout neurons reveals that neither spontaneous nor evoked release is affected by the absence of syntaxin-1a, although LTP induction was impaired with a decrease in monoaminergic transmission (Fujiwara et al., 2006; Mishima et al., 2012). This mild phenotype of Stx1a knockout mice might be stemming from genetic redundancy, where Stx1b is likely compensating the loss of Stx1a, considering their high homology. On the other hand, the loss of Stx1b was shown to have more severe outcomes than the loss of Stx1a alone, indicating that Stx1b is the dominant paralog in neuronal survival and function. Stx1b KO mice show severe impairments in motor coordination, and usually die by 2 weeks of age (Kofuji et al., 2014; Mishima et al., 2014; Figure 3B). Unlike Stx1a, the lack of Stx1b also affects the central nervous system development, and results in a smaller cerebellum size with decreased Purkinje cell arborization (Fujiwara et al., 2006; Kofuji et al., 2014). Neurons lacking Stx1b are more prone to degeneration and show decreased number of docked synaptic vesicles. The electrophysiological investigation of Stx1b knockout neurons revealed that evoked release is not significantly affected, shows only a small decrease in release probability, whereas spontaneous release frequency is significantly decreased accompanied by a decrease in RRP size (Mishima et al., 2014). Due to its critical role in neuronal survival and synaptic transmitter release, in this section, we focused on the reports characterizing pathogenic variants of Stx1b.

To date, there are 55 reported pathogenic (or likely pathogenic) Stx1b variants that comprise 13 frameshift (T285DfsX75, Q185DfsX8, A135GfsX52, E128RfsX2, L112CfsX18, A85SfsX7, V20WfsX34, D13KfsX42, S10AfsX7, Q52EfsX2, N189AfsX5, F215CfsX9, and Q56TfsX3), 15 missense (G275R, S239P, S239F, G226R, V216E, C144F, R115P, S98R, V88F, E210K, A246P, S258Q, R261Q, I282T, and S98N), 11 nonsense mutations (Y256X, R245X, Y234X, E132X, E96X, Q72X, S47X, K54X, Q56X, K93X, and Y140X), and two insertion/deletion (indel) mutations (K45indelRMCIE and I229DdelinsN), in addition to 14 mutations that result in splice variants, which alter RNA splicing and cause translation of an abnormal protein product that may contain intronic sequences or miss critical exonic sequences. Clinical reports show that patients that harbor Stx1b mutations commonly present with epilepsy, frequently with febrile seizures, and developmental delay (Figure 3A). The AED response is variable among patients, underlining variant-specific effects on the mechanisms of epileptogenesis (Schubert et al., 2014; Wolking et al., 2019; Verhage and Sørensen, 2020). Compared to the mutations of its binding partner Munc18-1, which results in DEEs with an early onset epilepsy (Ohtahara syndrome or West syndrome), missense mutations in Stx1b cause DEE which presents with a later onset epileptic disorder (Saitsu et al., 2008; Wolking et al., 2019; Krenn et al., 2021).

In 2014, the first pathogenic variants in the Stx1b gene were identified in families with fever-associated epilepsy syndromes that were diagnosed with genetic epilepsies with febrile seizures plus (GEFS+) or DEE, identified by whole exome sequencing (Schubert et al., 2014). In 2019, in a cohort of 46 individuals from 23 families that harbor Syntaxin-1b mutations, 17 new pathogenic Stx1b variants were identified. Among these 17 variants, there were 8 missense mutations (V88F, C144F, E210K, L221P, A246P, S258Q, R261Q, and I282T), 5 frameshift mutations (S10AfsX7, Q52EfsX2, E128GfsX2, N189AfsX5, and T285DfsX75), three nonsense mutations (K93X, Y140X, and R245X), and one patient with complete deletion of the Stx1b gene (Wolking et al., 2019). Upon characterization of different clinical phenotypes, authors categorized Stx1b-associated epileptic disorders into four subgroups depending on their clinical presentations and formed the following categories: 1. genetic epilepsy with febrile seizures plus (GEFS+) with a benign course, with a good anti-epileptic drug response, 2. genetic generalized epilepsy (GGE), and 3. developmental and epileptic encephalopathy (DEE) with moderate to severe developmental delay and 4-focal epilepsy. Among different pathogenic variants, *de novo* missense variants were associated with severe neurodevelopmental and epileptic phenotypes whereas nonsense (truncating) mutations that result in loss-of-function were associated with milder epileptic phenotypes. This could be explained by a dominant negative effect of a defective protein product as a result of missense mutations, as opposed to truncating nonsense mutations in Stx1b that likely undergoes nonsense-mediated decay, which could be compensated by Stx1a (Wolking et al., 2019).

To date, the number studies investigating the functional outcomes of specific Stx1b mutations is scarce. For the first time, Stx1b-V216E was expressed in Stx1b knockdown zebrafish larvae but failed to rescue the seizures induced by low levels of Stx1b expression, suggesting loss-of-function caused by this variant (Schubert et al., 2014; Figure 3C). In 2020, the functional implications of three Stx1b mutations (a complex insertion-deletion mutation K45/RMCIE; two missense mutations V261E and G226R) on synaptic vesicle release were investigated using primary neuron cultures. The insertion/deletion (indel) mutation (K45/RMCIE), which is in the H_*abc*_ domain, was found to destabilize the protein structure, abolish the binding of Stx1b to Munc18-1 and decrease both Stx1b and Munc18-1 levels. The indel mutation, K45/RMCIE, did not restore the abnormal synaptic transmission in Stx1-null neurons, showed no measurable evoked neurotransmitter release, RRP size or spontaneous release and could only partially rescue the deleterious effects of lack of Stx1 on neuronal survival. Although K45/RMCIE was able to form SNARE complexes *in vitro*, it was not able to mediate neurotransmitter release, most likely due to its instability and the absence of Munc18-1 binding (Vardar et al., 2020). Two other mutations in the SNARE motif, G226R and V216E could both rescue the lethal phenotype of Stx1-null neurons and were able to form SNARE complexes. Both G226R and V216E variants significantly affected the binding of Stx1b to Munc18-1 and Munc13 (Vardar et al., 2020) where the G226R variant resulted in a decreased binding to Munc18-1 but an augmented binding to Munc13 whereas the V216E variant resulted in a decreased binding to both Munc18-1 and Munc13. The G226R variant resulted in decreased evoked release, accompanied by a decrease in RRP size but did not affect spontaneous neurotransmitter release (Vardar et al., 2020). V216E variant, that is in the loop that regulates autoinhibitory regulation of Stx1b-Munc18-1 complex, did not significantly affect evoked release, spontaneous release or RRP size compared to WT controls but caused a decrease in paired pulse ratio (Vardar et al., 2020; Figure 3D). The variability of synaptic effects of Stx1b mutations could be due the fact that Stx1b being a major hub protein, which interacts with various other presynaptic proteins, such as Munc18-1 (Hata et al., 1993), N-type Ca^2+^ channels (Sheng et al., 1994) and complexins (Pabst et al., 2000) to regulate proper and synchronized synaptic vesicle release through its highly complex protein–protein interactions. Alterations in the structure of syntaxin-1 could result in disturbances in synaptic vesicle release due to alterations in one or several of these complex protein–protein interactions, resulting in an array of synaptic and cellular aberrancies. Considering the high number of variants in Stx1b that result in clinically significant phenotypes, there is need for further research to determine variant specific effects of Stx1b on neuronal and synaptic function. This would not only increase our understanding of the functions of Stx1b on different modes of synaptic vesicle release, but also could provide novel treatment targets that will benefit the patients suffering from Stx1b-associated epileptic diseases.

## 3. Synaptotagmin-1

Synaptotagmin-1 (Syt-1) is a low affinity calcium sensor that couples action potentials to the fast synchronous neurotransmitter release (Bowers and Reist, 2020; Figure 3E). Following the arrival of an action potential and consequential presynaptic calcium influx, Ca^2+^ binds to Syt-1 through its highly conserved C2A and C2B domains that results in an electrostatic switch and subsequent plasma membrane penetration *via* the hydrophobic residues within these domains (Bacaj et al., 2013; Südhof, 2013). This interaction results in zippering of the SNARE complex and synaptic vesicle exocytosis. In addition to its vital role in exocytosis, Syt-1 also plays modulatory roles in synaptic vesicle endocytosis (Zhang et al., 1994; Poskanzer et al., 2003; Yao et al., 2011; Li et al., 2017), is an important determinant of the synchronicity of neurotransmitter release (Uzay et al., 2023), plays a role in the maintenance of RRP and clamps spontaneous release of vesicles (Liu et al., 2009; Courtney et al., 2019). Syt-1 knockout mice are initially viable at birth but fail to perform coordinated behaviors such as suckling and Syt-1 deficient neurons show a significantly desynchronized evoked neurotransmitter release with decreased amplitude in addition to an increase in spontaneous vesicle fusion as a result of unclamping (Geppert et al., 1994; Littleton et al., 1994; Liu et al., 2009; Figure 3F).

To date, there are 15 reported pathogenic (or likely pathogenic) Syt1 variants which are all missense mutations that take place either in the C2A domain (4 variants; L159R, T196K, E209K, and E219Q) or more frequently in the C2B domain (11 variants; I368T, M303K, M303V, S309P, N341S, Y365C, D304G, D366E, N371K, G396D, and K367dup) (Figure 3E). Although both the C2B and C2A domains are structurally very similar, C2B domain is the dominant, highly energetic domain mediating membrane fusion (Gruget et al., 2020). In line with this, the most clinically consequential mutations in Syt-1 take place in the C2B domain (Baker et al., 2015, 2018). Mutations that neutralize C2B domain abolish evoked neurotransmitter release and Ca^2+^ dependent vesicle docking (Chang et al., 2018; Gruget et al., 2020), whereas mutations in the C2A only partially decrease neurotransmitter release and are better tolerated (Stevens and Sullivan, 2003; Paddock et al., 2011). Variants in the SYT1 gene give rise to a neurodevelopmental disorder, also known as Baker-Gordon syndrome (OMIM 618218), that commonly presents with infantile hypotonia, developmental delay, ID, ophthalmic abnormalities, and movement disorders. Although epilepsy is not a common presentation, seizures could be seen (Baker et al., 2015, 2018; Melland et al., 2022; Figure 3E).

In 2015, the first cases of neurodevelopmental disorders caused by pathogenic Syt-1 variants were reported in two patients harboring I368T and M303K variants. The patient harboring I368T variant presented with an early-onset mixed hyperkinetic movement disorder, severe motor delay, profound cognitive impairment and bilateral esotropia, and the patient that harbors M303K variant presented with various morphological abnormalities, developmental delay, esotropia, and EEG abnormalities without seizures (Baker et al., 2015; Cafiero et al., 2015). In 2018, three new *de novo* mutations (D304G, D366E, and N371K) were reported that have a similar clinical presentation as the previous reports, in addition to various behavioral abnormalities. In 2021, the phenotypic spectrum of Syt1-associated neurodevelopmental disorder was expanded, upon identification of various novel pathogenic (or likely pathogenic) variants, including the ones in the C2A domain (Melland et al., 2022). *In silico* molecular dynamics simulation of Syt-1 variants proposed distinct variant-specific perturbations in the structure and function of Syt-1. Variants could alter the regional mobility of their respective domains (L159R and T196K in C2A; Y395C, M303V, and K367dup in C2B), disturb intramolecular interactions (E209K, E219Q, and S309P), perturb SNARE binding (N341S which is at the spot that binds to SNAP25) or alter the overall surface charge of the protein (E219Q). In addition, some of the mutations in the C2B domain that alter the Ca^2+^-binding pockets could affect the calcium binding of Syt-1 which would impair proper synaptic vesicle fusion (Melland et al., 2022).

The severity of the dysfunction in the neurotransmitter release caused by different Syt-1 variants depends on the location of the amino acid substitution and its consequent pathogenic mechanism of action. D304G and D366E mutations take place in a residue that directly contacts calcium, therefore affecting calcium binding (Baker et al., 2018), whereas I368T occurs in a region that mediates Ca^2+^-dependent penetration of Syt-1 into the lipid membrane, where insertion of hydrophilic threonine impairs membrane penetration, therefore synaptic vesicle release (Baker et al., 2015). When expressed in wild-type mouse hippocampal neurons, D304G, D366E, I386T, and N371K variants can localize to nerve terminals at rest, resulting in impaired synaptic vesicle exocytosis, assessed by live cell imaging (Baker et al., 2018). The expression of M303K Syt-1 in wild-type mouse neurons, however, show that this variant does not localize to nerve terminals and results in a decrease in overall Syt1 expression. Following prolonged depolarization, I368T and N371K were able to be effectively retrieved to the presynaptic terminal whereas D304G and D366E fail to be retrieved, suggesting that these mutations impair endocytosis and vesicle recycling (Baker et al., 2018). When expressed in Syt1-knockout neurons, D304G, D366E, and I368T variants all show perturbed evoked neurotransmitter release, although having varying effects on spontaneous vesicle fusion. D366E and I368T variants were able to clamp spontaneous release, whereas D304G variant resulted in an increase in spontaneous vesicle release indicating loss-of function underscoring the role of Ca^2+^ binding loops in clamping of the spontaneous fusion (Bradberry et al., 2020; Figure 3G). When normal Syt-1 was co-expressed with the pathogenic variants, synaptic neurotransmitter release was restored which correlated with the mutant/healthy protein ratio (Bradberry et al., 2020). The disruptive effects of these variants were able to be mitigated by increasing presynaptic Ca^2+^, through a K^+^ channel blocker, 4-AP, that is an FDA-approved medication for the treatment of multiple sclerosis. Administration of 4-AP dose-dependently increased the evoked glutamate release in neurons expressing D304G, D366E, and I368T variants (Bradberry et al., 2020). This strategy has been also employed to overcome synaptic deficits associated with VAMP2 mutations (Simmons et al., 2020).

There is a substantial phenotypic heterogeneity among different variants suggesting varying degrees of allelic expressivity. These Syt1 variants could be differentially expressed compared to wild-type proteins in various different brain regions, neuron subtypes and synapse types, altering circuits, and impairing function (Baker et al., 2018; Verhage and Sørensen, 2020; Melland et al., 2022). The functional impact of each Syt-1 variant on different modes of neurotransmitter release needs to be investigated for thorough functional characterization of Syt-1 in human CNS function and disease.

## 4. Complexin

**FIGURE 4:**
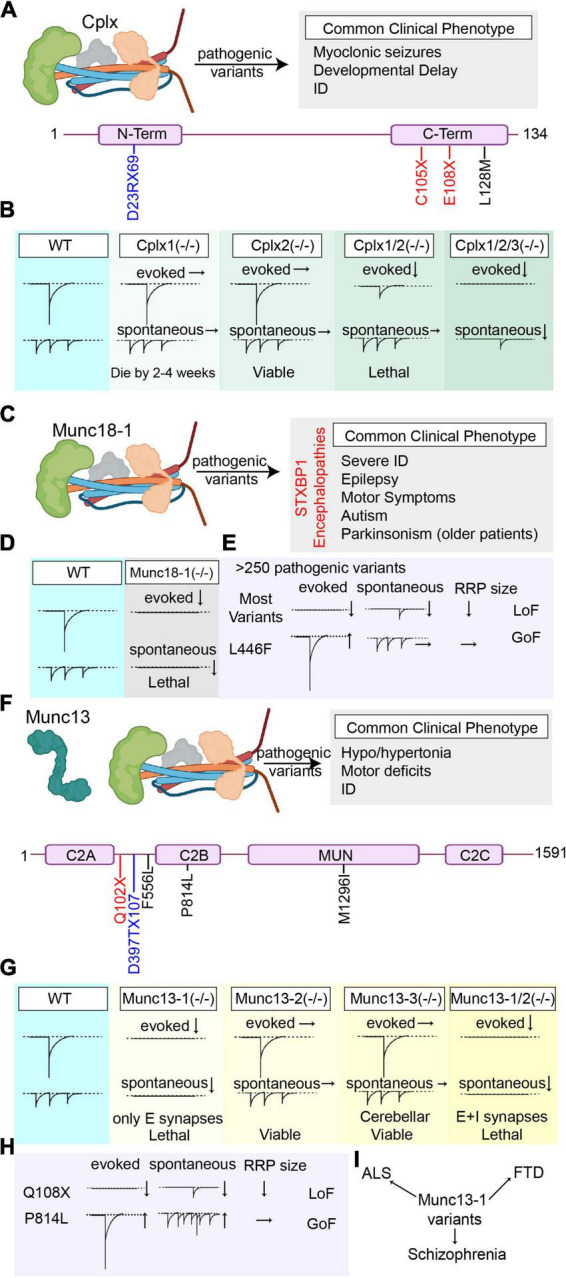
Overview of the pathogenic Cplx1, Munc18-1, and Munc13 variants’ effects on synaptic vesicle release. **(A)** Summary of all reported pathogenic Cplx1 (NCBI Accession #: NP_006642.1) variants and the most common clinical phenotype of the patients harboring these variants (Variants are color-coded based on their effects on the protein. Nonsense mutations are presented in red, missense mutations are presented in black, and frameshift mutations are presented in blue). **(B)** Graphical depiction demonstrating the changes in synaptic vesicle release upon genetic deletion of Cplx1 and/or Cplx 2 and upon triple knockout of Cplx1/2/3. **(C)** Summary of the most common clinical phenotype of the patients harboring pathogenic Munc18-1 (NCBI Accession #: NP_001361240.1) variants (STXBP1 encephalopathies). **(D)** Graphical depiction demonstrating the changes in synaptic vesicle release upon genetic deletion of Munc18-1. **(E)** Graphical summary of the findings explaining the loss-of-function outcomes of most Munc18-1 variants and the gain-of-function effects of L446F variant. **(F)** Summary of all reported pathogenic Munc13 (NCBI Accession #: AAC19406.1) variants and the most common clinical phenotype of the patients harboring these variants (Variants are color-coded based on their effects on the protein. Nonsense mutations are presented in red, missense mutations are presented in black, and frameshift mutations are presented in blue). **(G)** Graphical depiction demonstrating the changes in synaptic vesicle release upon genetic deletion of Munc13-1, Munc13-2, Munc13-3, and double knockout of Munc13-1/2. **(H)** Graphical summary of the findings explaining the effects of different Munc13 variants on different modes of neurotransmitter release (Q108X and P814L). **(I)** Munc13 variants are not only associated with early-onset neurobehavioral symptoms as the pathogenic variants in the other components of the release machinery, but also are associated with some complex diseases like ALS, FTS, and schizophrenia that develop later in life.

Complexins are soluble presynaptic proteins that stabilize SNARE complex formation by competing with α-SNAP, prevent depriming, and facilitate Ca^2+^-dependent vesicle fusion (McMahon et al., 1995; Reim et al., 2001; Figure 4A). So far, four main isoforms of complexins have been identified, which comprise complexin-1 (Cplx1), the major brain isoform that is generally co-expressed with complexin-2, in addition to complexin-3, and complexin-4, which are the isoforms more commonly expressed at retinal ribbon synapses (Reim et al., 2005). Complexin-2 knockout mice are viable and do not show a significant phenotype, whereas complexin-1 knockout mice are both ataxic and epileptic, and die in 2–4 weeks after birth, indicating that complexin-1 is critical for survival and proper functioning of the CNS. In line with these findings, complexin-1/2 double knockout mice are not viable and die a few hours after birth. Electrophysiological investigation of neurons lacking complexin-1 or complexin-2 alone do not show any differences in evoked or spontaneous vesicle release compared to WT controls, however the loss of complexin-1/2 together results in a significant decrease in evoked release, without affecting spontaneous release, RRP size or synapse density. Moreover, increasing the extracellular Ca^2+^ concentration was able to mitigate this defect in evoked release, indicating decreased Ca^2+^ sensitivity upon loss of complexin-1/2 (Reim et al., 2001). As an effort to circumvent genetic redundancy, complexin-1 was conditionally knocked out in a complexin-2/3 double knockout background and the acute triple knockout of complexin-1/2/3 resulted in a significant decrease in synchronous, asynchronous, and spontaneous neurotransmitter release, indicating that complexins facilitate all modes of vesicle fusion (Xue et al., 2008; López-Murcia et al., 2019; Figure 4B).

To date, only four Cplx1 pathogenic (or likely pathogenic) variants are reported, which comprise two nonsense mutations (E108X and C105X), one frameshift mutation (D23RfsX69) and one missense mutation (L128M) in the C domain of Cplx1. Patients commonly present with migrating myoclonic epilepsies, developmental delay and intellectual disability (ID) (Figure 4A). In 2017, the first Cplx1 variant was identified, homozygous E108X nonsense mutation, in a large cohort of patients upon whole exome sequencing (WES) on consanguine families with neurodegenerative disorders. Patients harboring the E108X variants presented with migrating malignant epilepsy, accompanied by cortical atrophy (Karaca et al., 2015). In 2019, additional variants were identified (C105X and L128M) that presented with myoclonic epilepsies that are resistant to AEDs, in addition to ID and developmental delay (Redler et al., 2017). Among these variants, L128M missense amino acid change takes place in the C-terminus of complexin-1, which is known to play a role in proper subcellular localization of complexin-1, in addition to regulating spontaneous vesicle fusion. Therefore, perturbations in the C-terminal could interfere with its proper localization in the presynaptic terminal, resulting in loss-of-function (Gong et al., 2016). So far, the functional implications of these variants on neurotransmitter release have not been studied, and how different Cplx1 variants result in a unique epileptic profile accompanied by developmental and intellectual disabilities is a critical question that warrants further investigation. This future research would increase our understanding of both the functions of complexin-1 in the CNS function and the complex pathophysiology of intellectual disabilities.

## 5. Munc18-1

Munc18-1, also known as the syntaxin binding protein 1 (STXBP1) is a key presynaptic protein that plays critical roles in synaptic vesicle fusion (Voets et al., 2001; Graham et al., 2004; Rizo and Xu, 2015; Figure 4C). Munc18-1 binds to syntaxin-1 and prevents formation of ectopic SNARE complexes during its trafficking to the cell surface and to the presynaptic membrane (Medine et al., 2007). The binding of Munc18-1 to syntaxin-1 establishes proper localization and conformation of syntaxin-1 before it assembles with SNAP25 to form the acceptor complex, rendering it easier for synaptotagmins to bind to the acceptor complex aiding vesicle docking (de Wit et al., 2009). In addition, Munc18-1 promotes SNARE assembly by acting as a template for the SNARE proteins and cooperates with Munc13 to prevent depriming of synaptic vesicles (Han et al., 2010; Meijer et al., 2012; Sitarska et al., 2017; Abramov et al., 2021; Tang et al., 2021).

Deletion of Munc18-1 completely abolishes neurotransmitter release without affecting the brain assembly during embryogenesis. Munc18-1 knockout die immediately after birth due to respiratory insufficiency (Verhage et al., 2000; Figure 4D). Although the brain assembly is not affected, loss of Munc18-1 leads to a widespread neuronal apoptosis and degeneration, underscoring its essential functions in neuronal survival in addition to synaptic vesicle fusion (Verhage et al., 2000; Feringa et al., 2023). Since the knockout of Munc18-1 is lethal, investigation of the loss-of-function effects of this protein in the central nervous system is only possible through the heterozygous Munc18-1 (+/−) mice. Munc18-1 (+/−) mouse models can strongly recapitulate the disease phenotype associated with STXBP1-encephalopathies, and result in 50% reduction in the Munc18-1 levels in most brain regions (Chen et al., 2020). These mice exhibit severe cognitive, motor dysfunctions, increased anxiety-like behavior, and epileptiform activity. Selective impairment in parvalbumin and somatostatin interneurons were found to be responsible from cortical hyperexcitability that results in epileptiform spike-wave discharges (Chen et al., 2020).

To date, more than 250 pathogenic (or likely pathogenic) Munc18-1 variants are reported, that comprise missense, nonsense, frameshift mutations, and various splice-site variants in addition to deletions. Patients harboring these mutations commonly present with severe intellectual disability, epilepsy, motor disturbances (hypotonia, dystonia, dyskinesia, and spasticism), and autistic behavior (Stamberger et al., 2016; Abramov et al., 2021; Figure 4C). Older patients with Munc18-1 mutations also show signs of Parkinsonism including tremor and bradykinesia (Keogh et al., 2015; Álvarez Bravo and Yusta Izquierdo, 2018).Co-aggregation of mutant Munc18-1 with α-synuclein is thought to be the underlying mechanism of parkinsonian symptoms that are seen in older patients (reviewed in Lanoue et al., 2019).

In 2008, the first pathogenic Munc18-1 variants were identified in five patients that suffer from an infantile epileptic encephalopathy, Ohtahara syndrome, which presented with intractable seizures accompanied by severe psychomotor retardation (Saitsu et al., 2008). This study was followed by a number of studies identifying numerous Munc18-1 mutations in patients suffering from not only early onset epileptic encephalopathies such as West syndrome, Lennox-Gastaut syndrome, and Dravet syndrome, but also in patients that present without an epileptic phenotype and are diagnosed with atypical Rett syndrome, intellectual disability without epilepsy, ataxia-tremor-retardation syndrome without epilepsy. This diagnostic odyssey, combined with high prevalence of STXBP1 mutations resulted in recategorization of STXBP1-associated syndromes to STXBP1-encephalopathies (Stamberger et al., 2016). STXBP-1 encephalopathies form a significantly large portion of all SNAREopathies and are thoroughly reviewed in a number of wonderful reviews (Saitsu et al., 2012; Stamberger et al., 2017; Abramov et al., 2021). Therefore, we did not include the effects of each Munc18-1 variant in this review and encourage readers to scrutinize these previous publications. In rest of this section, we mainly focus on the effects of some specific variants that result in gain-of-function effects, and the most recent efforts to develop treatment strategies for STXBP1 encephalopathies.

The majority of the variants in the STXBP1 gene result in a loss-of-function effect due to significantly decreased Munc18-1 levels upon degradation of the truncated or mutated unstable Munc18-1 protein, although some variants that result in gain-of-function effect are also reported (Saitsu et al., 2008; Guiberson et al., 2018; Kovačević et al., 2018; Chen et al., 2020; Lammertse et al., 2020). Moreover, the mutant STXBP1 can aggregate with wild-type STXBP1, promoting its degradation, therefore ending up having dominant negative effects (Guiberson et al., 2018). Munc18-1, similar to syntaxin-1, has many binding partners, and missense mutations in STXBP1 gene, depending on their location, could also perturb the binding of Munc18-1 to its interactors. This diverse interactome could result in diverse outcomes upon specific amino acid substitutions (Guiberson et al., 2018). For example, the missense mutation P335L results in a gain-of-function phenotype, due to a structural change that promotes SNARE complex formation, hence causes an increase in synaptic vesicle fusion (Guiberson et al., 2018). In a recent study, another missense mutation, L446F, was reported that results in a gain-of function phenotype, without significantly affecting Munc18-1 levels, and that significantly increases evoked neurotransmitter release without affecting spontaneous release or RRP size (Lammertse et al., 2020; Figure 4E). These findings alter the general paradigm in studying STXBP1 encephalopathies, where the significant loss of Munc18-1 is expected, and underscores the need for additional research to expand the efforts to understand the pathophysiology of STXBP1 encephalopathies.

Currently, the treatment options for STXBP1 encephalopathies are scarce and are mainly focused on the seizure management, where drug resistance is very common (Stamberger et al., 2016). To prevent Munc18-1 aggregation and consequent degradation, three chemical chaperones, trehalose, sorbitol an 4-phenylbutyrate (4-PB) were proposed as potential therapeutics, that were able to restore Munc18-1 protein levels and rescue synaptic deficits (Guiberson et al., 2018; Abramov et al., 2021) by preventing unfolded protein aggregation and degradation (Cortez and Sim, 2014). Among these chaperones, 4-PB is particularly promising, since it crosses blood-brain barrier, and is already FDA-approved for treatment of urea-cycle disorders (Maestri et al., 1996; Iannitti and Palmieri, 2011). In 2021, the first clinic pilot trial (Phase 1 study) of 4-PB in a group of 21 STXBP1 encephalopathy patients was started to test the use of 4-PB as a therapeutic option in this patient population (Clinical Trial Identifier: NCT04937062). The heterozygous mutations that cause a decrease in WT Munc18-1 levels and end up in loss-of-function could benefit from this therapy, however mutations like L446F or P335L, that result in a gain-of-function would not be good candidates. Additional studies are to be performed to develop treatment targets to alleviate the outcomes of different variants that result in gain-of-function effects, to efficaciously treat different types of STXBP1 encephalopathies.

## 6. Munc13

Munc13 is a critical component of synaptic vesicle priming machinery that modulates efficient SNARE complex formation during synaptic vesicle release. Munc13 binds to the Munc18-syntaxin complex through its MUN domain and results in a configurational change that opens syntaxin-1 for SNAP25 binding (Jahn and Fasshauer, 2012; Figure 4F). There are three paralogs of Munc13 in the central nervous system. Among these, Munc13-1 forms the dominant paralog in multiple regions of brain, which is co-expressed with Munc13-2, whereas Munc13-3 is almost exclusively expressed in the cerebellum (Augustin et al., 1999, 2001). Munc13-1 knockout mice die shortly after birth whereas Munc13-2 or 13-3 knockout mice are viable and do not show a significantly different phenotype. Munc13-3 knockout mice only demonstrate disrupted motor learning, associated with decreased release probability in parallel fiber-Purkinje cell synapses indicating affected cerebellar functions (Augustin et al., 1999; Varoqueaux et al., 2002). Neurons that lack Munc13-1 show a significantly decreased evoked and spontaneous release accompanied by a decrease in RRP size in the glutamatergic synapses without affecting GABA-mediated neurotransmission, whereas the knockout of Munc13-3 or Munc13-2 alone do not have significant effects on synaptic vesicle fusion (Augustin et al., 1999; Varoqueaux et al., 2002). When Munc13-2, the only Munc13 paralog that is co-expressed with Munc13-1 in the hippocampus, and Munc13-1 are knocked out together, neurons show complete cessation of spontaneous and evoked neurotransmitter release both in excitatory and inhibitory synapses in addition to a large reduction in docked vesicles (Varoqueaux et al., 2002; Imig et al., 2014; Figure 4G). Despite this significant impairment in neurotransmitter release, embryonic development of brain and the spinal cord stays unaffected (Varoqueaux et al., 2002).

To date, at least five pathogenic (or likely pathogenic) variants are reported in the Munc13-1 protein, which comprise one nonsense mutation (Q102X) that results in a loss of function phenotype, three missense mutations (P814L, M1296I, and F556L) and one frameshift mutation (D397TfsX107). In 2016, the first Munc13-1 variant, Q102X, was identified that results in early truncation of the protein, most likely resulting in a loss-of-function effect (Figure 4F). The patient harboring the Q102X variant presented with microcephaly, cortical excitability, myasthenia, severe hypotonia with motor defects, and died due to respiratory insufficiency at 4 years of age (Engel et al., 2016). Electrophysiological investigation of the muscle sample of this patient showed severely reduced spontaneous and evoked release events. Ultrastructural investigation showed that although the motor endplate architecture was not affected, the RRP size was significantly decreased (Engel et al., 2016). In 2017, a missense mutation in the Munc13-1, P814L, which results in gain-of-function effects, was identified. The patient harboring the P814L variant presented with ID, dyskinesia, autism, ADHD and delayed neurological development. Electrophysiological investigation of the P814L variant using primary neuron cultures showed that this variant significantly increases both evoked and spontaneous vesicle fusion without changing the RRP size (Lipstein et al., 2017; Figure 4H). This variant was shown to increase release probability and caused rapid synaptic depression upon high frequency stimulation indicating increased fusogenicity of the synaptic vesicles. This increase in vesicle fusion propensity was hypothesized to be due to increased activity of presynaptic voltage gated Ca^2+^ channels that leads to an increased Ca^2+^ influx, boosting vesicle fusion. This premise warrants further research to understand how this variants results in augmented vesicle fusion (Lipstein et al., 2017). In a recent study, a frameshift mutation, D397TfsX107, was reported in a patient that presented with congenital encephalopathy accompanied by facial dysmorphisms, alternating hypotonia/hypertonia, infantile spasms, and died at 8 months of age. Postmortem investigation of patient’s brain showed blunted frontal and temporal lobes, cerebellar gliosis, abnormal hippocampus positioning, and axonal spheroids in the septum pellucidum (Mullins et al., 2022). These studies indicate that Munc13-1 has diverse functions regarding proper CNS development and function, and changes in the Munc13-1 structure could not only significantly perturb synaptic vesicle release but also completely alter normal brain architecture.

Apart from these point mutations that result in congenital encephalopathies with neurodevelopmental outcomes, Munc13-1 variants are also reported in sporadic amyotrophic lateral sclerosis (ALS) (F556L), frontotemporal dementia (FTD) and schizophrenia (M1296I) patients (Fromer et al., 2014; Placek et al., 2019; Pottier et al., 2019; Brown et al., 2022; Figure 4I). These findings point out that the functions of Munc13-1 is not only restricted to early development of the CNS but also could significantly alter the CNS homeostasis later in life. How these specific variants affect different CNS parts and result in sporadic complex neuropsychiatric phenotypes in the adulthood is a conundrum that demands further research.

## 7. Discussion

In the last two decades, hundreds of variants were identified in the core components of synaptic vesicle release machinery that result in severe neurological and neuropsychiatric phenotypes. The clinical assessment of the patients harboring these mutations complements the efforts to determine the roles of release machinery components in CNS (patho-)physiology, not only cause early-onset epileptic encephalopathies but also for complex and more prevalent diseases like autism, schizophrenia, or ALS. Despite the advances in our understanding of how synaptic release machinery functions, there is a growing need to investigate how each of these pathogenic variant affects different modes of synaptic neurotransmitter release to determine novel treatment targets, personalized to specific variants and their consequent dysfunctions in the neural circuitry.

The most recent efforts to characterize the outcomes of different pathogenic variants of vesicle release machinery showed that evoked neurotransmitter release in not the only affected component, and aberrancies in spontaneous transmission alone can severely affect the CNS function (Alten et al., 2021). Although the ubiquitous role of spontaneous transmission in CNS function has been thoroughly investigated in the last decades, clinical guidelines and treatment options are mainly focused on pharmacotherapies targeting evoked neurotransmitter release, thereby not always providing sufficient treatment. Electrophysiological assessment of pathogenic variants in different components of the neurotransmitter release machinery and their effect on spontaneous vesicle fusion, particularly, is critical to fully elucidate the functional implications of these mutations, and to develop targeted treatment options. Spontaneous neurotransmission could be regulated by homeostatic plasticity mechanisms and could be effectively modulated by different pharmacotherapeutics (reviewed in Kavalali and Monteggia, 2020). The use of agents that target homeostatic plasticity mechanisms in the treatment of SNAREopathies that selectively perturb spontaneous transmission could open up new venues both in our understanding of how the different components of the vesicle release machinery functions in the CNS and in the clinical management of the SNAREopathies.

Similar to SNAREopathies, pathogenic variants in the voltage-gated K^+^, Na^+^, and Ca^2+^ channels (known as channelopathies), result in a parallel pattern of symptom diversity with varying severity (Kullmann, 2010). A number of pathogenic variants have been identified in the genes encoding voltage-gated ion channels that gives rise to neurodevelopmental disorders that could present with ataxia, epilepsy, and intellectual disability (Jaudon et al., 2020). The similarity in clinical presentation indicates aberrant neurotransmission in channelopathies and underscores the need for a thorough electrophysiological investigation to determine the changes that take place in different modes of neurotransmission.

## Author contributions

BU and EK performed the writing and editing of the manuscript. Both authors contributed to the article and approved the submitted version.
